# Mortality and associated factors among people living with HIV admitted at a tertiary-care hospital in Uganda: a cross-sectional study

**DOI:** 10.1186/s12879-024-09112-7

**Published:** 2024-02-22

**Authors:** Darius Owachi, Praise Akatukunda, Diana Sarah Nanyanzi, Rogers Katwesigye, Shardrack Wanyina, Martin Muddu, Samuel Kawuma, Nelson Kalema, Charles Kabugo, Fred C. Semitala

**Affiliations:** 1 Kiruddu National Referral Hospital, Kampala, P.O. BOX 6588, Uganda; 2 Makerere University Joint AIDS Program, Kampala, Uganda; 3 Infectious Diseases Institute, Kampala, Uganda; 4 Department of Medicine, Makerere University, Kampala, Uganda

**Keywords:** Advanced HIV disease, Mortality, Inpatient, Hospitalization, Uganda

## Abstract

**Background:**

Hospital admission outcomes for people living with HIV (PLHIV) in resource-limited settings are understudied. We describe in-hospital mortality and associated clinical-demographic factors among PLHIV admitted at a tertiary-level public hospital in Uganda.

**Methods:**

We performed a cross-sectional analysis of routinely collected data for PLHIV admitted at Kiruddu National Referral Hospital between March 2020 and March 2023. We estimated the proportion of PLHIV who had died during hospitalization and performed logistic regression modelling to identify predictors of mortality.

**Results:**

Of the 5,827 hospitalized PLHIV, the median age was 39 years (interquartile range [IQR] 31–49) and 3,293 (56.51%) were female. The median CD4 + cell count was 109 cells/µL (IQR 25–343). At admission, 3,710 (63.67%) were active on antiretroviral therapy (ART); 1,144 (19.63%) had interrupted ART > 3 months and 973 (16.70%) were ART naïve. In-hospital mortality was 26% (1,524) with a median time-to-death of 3 days (IQR 1–7). Factors associated with mortality (with adjusted odds ratios) included ART interruption, 1.33, 95% confidence intervals (CI) 1.13–1.57, *p* 0.001; CD4 + counts ≤ 200 cells/µL 1.59, 95%CI 1.33–1.91, *p* < 0.001; undocumented CD4 + cell count status 2.08, 95%CI 1.73–2.50, *p* < 0.001; impaired function status 7.35, 95%CI 6.42–8.41, *p* < 0.001; COVID-19 1.70, 95%CI 1.22–2.37, *p* 0.002; liver disease 1.77, 95%CI 1.36–2.30, *p* < 0.001; co-infections 1.53, 95%CI 1.32–1.78, *p* < 0.001; home address > 20 km from hospital 1.23, 95%CI 1.04–1.46, *p* 0.014; hospital readmission 0.7, 95%CI 0.56–0.88, *p* 0.002; chronic lung disease 0.62, 95%CI 0.41–0.92, *p* 0.019; and neurologic disease 0.46, 95%CI 0.32–0.68, *p* < 0.001.

**Conclusion:**

One in four admitted PLHIV die during hospitalization. Identification of risk factors (such as ART interruption, function impairment, low/undocumented CD4 + cell count), early diagnosis and treatment of co-infections and liver disease could improve outcomes.

## Introduction

Access to antiretroviral therapy (ART) has significantly decreased the global deaths associated with the Human Immunodeficiency virus (HIV) infection. More than 29 million people living with HIV (PLHIV) were on ART by 2022, with HIV-associated deaths averted by up to 69% since the peak in 2004 [1]. Despite the improved access to ART, 630,000 global HIV-associated deaths were reported in 2022 with three in five deaths occurring in the African region [1]. HIV-associated mortality amongst hospitalized PLHIV in Africa is high, ranging between 13.6 and 38% [2–5]. Factors such as undiagnosed HIV status [6], low CD4 + cell counts [7, 8], tuberculosis [9], and co-infections [2–4] are among the common predictors of mortality among hospitalized PLHIV.

Uganda has made significant strides towards achieving the 95-95-95 HIV care cascade targets with more than 90% of PLHIV aware of their HIV status and 85% accessing ART [10]. However, despite these gains, an estimated 54,000 new HIV infections and 17,000 HIV-associated deaths were reported in 2022 [10]. There is a paucity of data on the factors associated with mortality among hospitalized PLHIV in Uganda. A recent study found that HIV disease was one of the leading causes of mortality among patients admitted at a major tertiary hospital in Uganda but did not explore the factors that predicted mortality in the HIV subpopulation [11]. An earlier study done before the test-and-treat era found an association between opportunistic infections and mortality among hospitalized PLHIV [12].

In March 2020, Makerere University Joint AIDS Program (MJAP) and Kiruddu National Referral Hospital (KNRH) partnered with the Infectious Diseases Institute (IDI) to establish a pilot program to support care for hospitalized PLHIV at KNRH. This was part of the Kampala HIV project run by the IDI with funding from the President’s Emergency Plan for AIDS Relief (PEPFAR), through the Centres for Disease Control and Prevention (CDC) [13]. The program aimed to reduce HIV-associated morbidity and mortality through the provision of extended support services for hospitalized PLHIV. The services offered included targeted HIV screening at the emergency department, diagnosis and treatment of opportunistic infections (OIs) and comorbidities, ART initiation and support counselling services, post-discharge counselling, and post-discharge linkage to care, as well as training of KNRH healthcare providers in managing HIV disease. These activities were based on the 2020 Ugandan National HIV Care and Treatment guidelines [14] and were integrated into the routine hospital procedures of KNRH. We analyzed the in-hospital mortality rate and predicting clinical and demographic factors among PLHIV admitted to KNRH between March 2020 and March 2023.

## Methods

### Study design and setting

This cross-sectional study was conducted at KNRH, a national referral hospital with a bed capacity of 200 and serves more than 100,000 patients annually [15] The hospital offers a range of medical and diagnostic services to the catchment population and established the first elaborate support program for hospitalized PLHIV in the country [13, 15].

### Study population

The study population included all identified PLHIV aged ≥ 12 years who were hospitalized at KNRH between March 2020 and March 2023.

### Sampling methods

All identified PLHIV admitted at KNRH between March 2020 and March 2023 were included in the study.

### Study procedure

We extracted, cleaned and entered de-identified data of patient health records into a password protected backed-up electronic Excel database. The clinical endpoint was the patient’s vital status “Alive” or “Dead” outcome at discharge/transfer from KNRH. Data on independent social-demographic variables included age, sex and distance of patients’ home addresses from KNRH estimated in kilometres (km) using arbitrary cut-offs of < 10 km, 10–20 km and > 20 km. clinical data variables included CD4 + cell counts, viral load, ART history (naïve or experienced at admission, adherence counselling), clinical diagnoses and function assessment scores (using the Eastern Cooperative Oncology Group (ECOG) score). ECOG scores of 3–4 were categorized as “poor function status”, indicating severe function impairment, while ECOG scores of 1–2 were categorized as “good function status” indicating mild to moderate function impairment).

Clinical diagnoses were categorized as “opportunistic infections” or “comorbidities.” The opportunistic infections (OIs) included TB, Cryptococcal disease, candidiasis, toxoplasmosis, and Kaposi sarcoma. The comorbidities included cardiovascular diseases, diabetes mellitus, kidney disease, liver disease, neurologic disorders, chronic lung diseases, cancers, anaemia and other co-infections (excluding OIs). Table 1 summarizes the definitions and categorization of the clinical diagnoses.

**Table 1:** Definitions and categorization of clinical diagnoses amongst hospitalized PLHIV at Kiruddu hospital

Tuberculosis	• Active disease considered when either of criteria was met: i. Bacteriologic confirmation of TB disease e.g., positive urine LAM, microscopy or GeneXpert ii. Clinical diagnoses made by an infectious disease consultant. iii. Patient taking anti-TB medication at the time of admission.
Cryptococcal disease	• Considered when either meningeal or non-meningeal disease forms were confirmed by positive cryptococcal antigen test or microscopy.
Candidiasis	• Either oral or oesophageal disease forms were considered, when diagnosed by clinical team
Toxoplasmosis	• Considered when diagnosed by clinical team based on radiologic and serologic evidence of toxoplasma infection.
Kaposi sarcoma	• Considered based on histopathologic diagnosis
COVID-19	• Considered when confirmed by a polymerase chain reaction test or rapid antigen diagnostic test
Co-infection	• Any diagnosis of an “infection” that excluded TB, cryptococcal disease, candidiasis, toxoplasmosis, and COVID-19. • Examples included diagnoses of respiratory tract infections (e.g., pneumonia), malaria, meningitis (bacterial, viral, or other), gastrointestinal infections, acute viral hepatitis infections, urine tract infections, skin infections, bloodstream infections and sepsis.
Cardiovascular disease	• Included diagnoses involving the cardiovascular system. • Examples included *hypertension* (patient with systolic blood pressure ≥ 140 mmHg or diastolic blood pressure ≥ 90 mmHg or when patient was on anti-hypertensive medication); any *cardiac disease* confirmed by cardiologist or echocardiography or other cardiac investigations; *peripheral artery disease, heart failure, cerebrovascular accidents* (stroke), and *venous thromboembolism.*
Malnutrition	• Considered where undernutrition was confirmed by anthropometric measurement (e.g., mid-upper arm circumference ≤ 22 mm or body mass index ≤ 18.5 kg/m^2^), or clinical evidence of wasting.
Anaemia	• Considered when measured haemoglobin concentration was < 12.9 g/dl in males or < 11.9 g/dl in females.
Diabetes Mellitus	• Considered when there was confirmed glycated haemoglobin ≥ 6.5% or random blood sugar ≥ 11mmol/L in the presence of symptoms of diabetes or when the patient was taking anti-diabetic medication at the time of admission.
Kidney disease	• Considered when there was evidence of elevated serum creatinine > 105µmol/L or urine output < 0.5 millilitres per kilogram bodyweight per hour, and a diagnosis of either acute kidney injury or chronic kidney disease made by clinical team.
Liver disease	• Considered where there was evidence of chronic viral hepatitis or chronic liver disease (e.g., cirrhosis or fibrosis), drug induced hepatitis or other forms of non-infectious hepatitis, liver failure or other forms of liver disease as determined by the clinical team.
Neurologic disorders	• Considered where diagnoses of epilepsy, seizure disorders or mental/psychiatric disorders were made following the exclusion of CNS infections.
Chronic lung disease	• Considered where a diagnosis of asthma, chronic obstructive pulmonary disease, interstitial lung disease, sequelae of post-TB infections in the absence of active TB disease, or other causes of chronic pulmonary disease, was made.
Cancers	• Considered where there was histopathologic confirmation of an oncologic diagnosis (excluding *Kaposi sarcoma*) or when the patient was on cancer therapy.

ART status at admission was categorized as “Active,” “Interrupted” or “Naïve” as guided by the 2020 National treatment guidelines. Patients who had never received ART (whether newly diagnosed or previously known HIV) were categorized as “ART Naïve” while a patient who interrupted ART ≥ three months before the current hospital admission was considered to have “Interrupted ART”. Patients were considered “Active on ART” if they or their attendants reported consistent use of ART regardless of duration at admission or if they had interrupted ART for a period < three months before admission.

### Statistical analysis

The primary outcome, mortality, was determined as the number of PLHIV who died divided by the total number of PLHIV that were admitted. Univariate analysis was performed to summarize the baseline characteristics of the study participants. For bivariate analysis, we used the Pearson chi-square test to determine the association between mortality and patients’ baseline characteristics. Variables with a *p* value greater than an arbitrary value of 0.25 were excluded from the multivariate analysis.

At multivariate analysis, adjusted odds ratios (AOR) from a multivariate binary logistic regression model were used with two-sided *p* values < 0.05 considered statistically significant. We performed analysis of variance between independent variables and excluded all variables which were highly correlated with each other, to control for type II errors. Multicollinearity was assessed using the Variance Inflation Factor (VIF) of dummy variables and the mean VIF was 1.36. Goodness of fit was performed after running the binary logistic regression model and was found to be correctly specified at a *p* value of 0.045. The model assumed characteristics of independence of independent variables, and non-inflated standard errors. Data analysis was done using Stata/MP 14 (Stata Corp LLC, Texas, USA).

### Ethical approval

Ethical approval was obtained from the Makerere University, College of Health Sciences, School of Public Health, research ethics committee under Reference MakSPH-REC710 and reference HS553ES under the Uganda National Council for Science and Technology. A waiver for informed consent was obtained since we sought to use only already existing routinely collected, de-identified program data requiring no contact with participants.

## Results

### Baseline characteristics

Of the 30,537 persons admitted at KNRH from March 2020 to March 2023, 5,827 (19.1%) PLHIV were identified, of whom 3,293 (56.5%) were females. The median age was 39 years (IQR 31–49 years), and the median duration of hospitalization was 5 days (IQR 2–10 days). CD4 + cell count was documented among 3,715 (63.8%) hospitalized PLHIV of whom 2,271 (38.97%) had CD4 + cell count ≤ 200 cells/µL. The median CD4 + cell count was 109 cells/µL (IQR 25–343 cells/µl).

A total of 4,854 (83.3%) PLHIV had initiated ART before admission of whom 3,710 (63.7%) were active on their treatment while 1,144 (19.6%) had interrupted ART longer than three months; 973 (16.7%) were ART naïve. A poor ECOG function assessment (ECOG score 3–4) was observed in 2,225 (38.2%) of hospitalized PLHIV. Table 2 summarizes the baseline characteristics of the patients at admission.

**Table 2:** Baseline characteristics for HIV + patients admitted at KNRH (March 2020– March 2023)

Variable	Discharged (*N* = 4,303)	Died (*N* = 1,524)	Total (*N* = 5,827)	*p* value
**Median Age** (IQR)	39(31–48)	40(31–50)	39 (31–49)	
**Age group** (years) (%)				
<24	333 (7.74)	101 (6.63)	434 (7.45)	0.053
25–49	2,976 (69.16)	1,029 (67.52)	4,005 (68.73)	
≥50	994 (23.10)	394 (25.85)	1,388 (23.82)	
**Sex**				
Female	2,483 (57.70)	810 (53.15)	3,293 (56.51)	0.002
Male	1,820 (42.30)	714 (46.85)	2,534 (43.49)	
**Address**				
Distance < 10 km from the hospital	1,720 (39.97)	550 (36.09)	2,270 (38.96)	0.001
Distance 10-20 km from the hospital	1,550 (36.02)	537 (35.24)	2,087 (35.82)	
Distance > 20 km from the hospital	1,033 (24.01)	437 (28.67)	1,470 (25.23)	
**Admission status**				
New admission	3,790 (88.08)	1,390 (91.21)	5,180 (88.90)	0.001
Readmission	513 (11.92)	134 (8.79)	647 (11.1)	
**ART Status at Admission**				
Active on ART	2,824 (65.63)	886 (58.14)	3,710 (63.67)	< 0.001
ART Naïve	717 (16.66)	256 (16.80)	973 (16.70)	
ART Interruption	762 (17.71)	382 (25.07)	1,144 (19.63)	
**Median Days of Hospital Stay** (IQR)	6 (3–11)	3 (1–7)	5 (2–10)	
**Median Viral Load** (*n* = 216)	50 (0–13,900)	95(0-119,000)	50(0–28,290)	
**Median CD4 cell count** (µL, IQR)	133 (30–380)	57 (16–197)	109 (25–343)	
**CD4 + cell counts**				
CD4 + ≤ 200 cells/µL	1,624 (37.74)	647 (42.45)	2,271 (38.97)	< 0.001
CD4 + > 201 cells/µL	1,189 (27.63)	255 (16.73)	1,444 (24.78)	
CD4 + Not Documented	1,490 (34.63)	622 (40.81)	2,112 (36.25)	
**Function Assessment**				
ECOG Score 1–2	3,175 (73.79)	427 (28.02)	3,602 (61.82)	< 0.001
ECOG Score 3–4	1,128 (26.21)	1,097 (71.98)	2,225 (38.18)	
**Clinical Diagnoses**				
Tuberculosis	1,429 (33.21)	525 (34.45)	1,954 (33.53)	0.378
Co-infections	910 (21.15)	457 (29.99)	1,367 (23.46)	< 0.001
Cardiovascular disease	757 (17.59)	218 (14.30)	975 (16.73)	0.003
Malnutrition	475 (11.04)	332 (21.78)	807 (13.85)	< 0.001
Cryptococcal Disease	558 (12.97)	181 (11.88)	739 (12.68)	0.271
Anaemia	454 (10.55)	192 (12.60)	646 (11.09)	0.029
Kidney Disease	338 (7.85)	176 (11.55)	514 (8.82)	< 0.001
Candidiasis	331 (7.69)	112 (7.35)	443 (7.60)	0.664
Diabetes Mellitus	304 (7.06)	86 (5.64)	390 (6.69)	0.057
Liver Disease	214 (4.97)	124 (8.14)	338 (5.80)	< 0.001
Neurologic disorders	191 (4.44)	38 (2.49)	229 (3.93)	0.001
Chronic Lung Disease	148 (4.04)	37 (2.86)	185 (3.73)	0.053
COVID-19	114 (2.65)	77 (5.05)	191 (3.28)	< 0.001
Cancer	108 (2.51)	39 (2.56)	147 (2.52)	0.916
Toxoplasmosis	102 (2.37)	40 (2.62)	142 (2.44)	0.580
Kaposi Sarcoma	91 (2.11)	33 (2.17)	124 (2.13)	0.906

ART - Antiretroviral therapy; ECOG - Eastern Cooperative Oncology Group; IQR– Inter quartile range; KNRH– Kiruddu National Referral Hospital

### Co-infections and comorbidities

The OIs included TB (1954, 33.53%), Cryptococcal disease (739, 12.68%), candidiasis (443, 7.6%), toxoplasmosis (142, 2.44%) and Kaposi sarcoma (124, 2.13%). Other comorbid diagnoses included co-infections (1,367, 23.46%), cardiovascular disease (975, 16.7%), severe malnutrition (807, 13.9%), anaemia (646, 11.1%) and kidney disease (514, 8.82%). (Table 2).

### In-Hospital mortality

A total of 4,303 (73.85%) PLHIV were discharged from the hospital with a median duration of hospitalization of 6 days (IQR 3–11 days), while 1,524 (26.15%) died with a median time to death of 3 days (IQR 1–7 days).

### Factors Associated with HIV Mortality

At bivariate analysis, the factors that had a statistically significant association with mortality included age, sex, distance between home address and KNRH, readmission status, ART status, CD4 + cell counts, function assessment, COVID-19, cardiovascular disease, kidney disease, liver disease, anaemia, co-infections, malnutrition, diabetes mellitus, neurologic disorders and chronic lung disease (Table 2).

At multivariate analysis, factors that predicted mortality included function impairment, CD4 + count ≤ 200 cells/µL, undocumented CD4 + status, address > 20 km from hospital, ART interruption, COVID-19, liver disease and co-infections. Factors that were protective of mortality included hospital readmission, chronic lung disease and neurologic disorders (Table 3). Malnutrition, kidney disease, cardiovascular disease, anaemia and age were excluded from the multivariate analysis due to high multicollinearity.

**Table 3:** Multivariate analysis model for predictors of mortality among hospitalized PLHIV at KNRH

Variable	Unadjusted Odds Ratio (95% CI)	*p* value	Adjusted Odds Ratio (95%CI)	*p* value
**Sex**				
Female	1		1	
Male	1.20 (1.07–1.35)	0.002	1.13 (0.99–1.29)	0.064
**Address**				
Distance < 10 km from hospital	1		1	
Distance 10-20 km from hospital	1.08 (0.94–1.24)	0.253	0.99 (0.85–1.15)	0.889
Distance > 20 km from hospital	1.32 (1.14–1.53)	< 0.001	**1.23 (1.04–1.46)**	**0.014**
**Admission Status**				
New	1		1	
Readmission	0.71 (0.58–0.87)	0.001	**0.70 (0.56–0.88)**	**0.002**
**ART Status at Admission**				
Active on ART	1		1	
ART Naïve	1.14 (0.97–1.34)	0.116	1.03 (0.85–1.23)	0.787
ART Interruption	1.60 (1.38–1.85)	< 0.001	**1.33 (1.13–1.57)**	**0.001**
**CD4 Documentation status**				
CD4 + > 201 cells/µL	1		1	
CD4 + ≤ 200 cells/µL	1.88 (1.59–2.21)	< 0.001	**1.59 (1.33–1.91)**	**< 0.001**
CD4 + Not Documented	1.96 (1.66–2.32)	< 0.001	**2.08 (1.73–2.50)**	**< 0.001**
**Function Assessment**				
ECOG Score 1–2	1		1	
ECOG Score 3–4	7.23 (6.34–8.24)	< 0.001	**7.35 (6.42–8.41)**	**< 0.001**
**Clinical Diagnoses**				
Liver disease	1.69 (1.35–2.13)	< 0.001	**1.77 (1.36–2.30)**	**< 0.001**
COVID-19	1.96 (1.46–2.63)	< 0.001	**1.70 (1.22–2.37)**	**0.002**
Co-infections	1.60 (1.40–1.82)	< 0.001	**1.53 (1.32–1.78)**	**< 0.001**
Diabetes Mellitus	0.79 (0.61–1.01)	0.057	0.82 (0.62–1.08)	0.157
Chronic lung disease	0.70 (0.48–1.01)	0.054	**0.62 (0.41–0.92)**	**0.019**
Neurologic disorders	0.55 (0.39–0.78)	0.001	**0.46 (0.32–0.68)**	**< 0.001**

ART - Antiretroviral therapy; ECOG - Eastern Cooperative Oncology Group; CI– Confidence intervals; KNRH– Kiruddu National Referral Hospital

## Discussion

In this study, we assessed in-hospital mortality and associated factors among hospitalized PLHIV in Uganda. In this young population with a median age of 39 years, the in-hospital mortality was 26%. Almost two-fifths of the hospitalized PLHIV had advanced HIV disease (CD4 + cell count ≤ 200 cells/µL) and more than a third were not on ART at the time of admission. Factors which predicted in-hospital mortality included ART interruption, CD4 + cell counts ≤ 200 cells/µL, undocumented CD4 + cell counts, function impairment, co-infections, liver disease and long distance from the hospital.

The high mortality observed in our study is of significant concern as over a quarter of the hospitalized PLHIV succumbed to their illness in an era of Universal Test and Treat [16]. This observation is similar to the high in-hospital mortality reported in previous studies, ranging between 14 and 38% across various African settings [2–4]. Notably, we observed that most of the deaths occurred within a week of hospitalization which echoes findings from a study in Sierra Leone [3]. Our study site being a tertiary-level health facility raises concerns over patient-related or health system-related factors that contribute to poor healthcare-seeking behaviour and late hospital presentation. The high proportion of hospitalized PLHIV presenting with advanced HIV disease (39%) and significant function impairment (38%) in our study suggests a delayed or late presentation for healthcare [17–19]. Health system barriers such as long distance to the hospital could be another contributing factor to poor outcomes among hospitalized PLHIV. We observed that PLHIV who lived > 20 km from the hospital had higher odds of death compared to those who stayed < 10 km from the hospital. This observation agrees with other studies that identified long distance to a health facility as a barrier to accessing healthcare, potentially increasing the risk of mortality [20]. Use of patient-centred differentiated care approaches that take HIV services closer to the PLHIV may help address such barriers [21]. Encouraging early health-seeking behaviour among PLHIV could also improve hospital outcomes.

Of note, more than a third of the hospitalized PLHIV in our study were not on ART (Naïve or interrupted) at the time of admission. We observed that those who had interrupted their ART had 1.3 times the odds of dying during hospitalization compared to those who were active on ART. Our observations agree with other community-based studies that showed that up to a third of PLHIV who disengaged from HIV care were at a higher risk of mortality [22–25]. Since suboptimal ART coverage is associated with poor survival outcomes, there is an urgent need to understand and address the factors that contribute to patient disengagement from HIV care. Examples of factors that propagate patient disengagement from care include transportation costs [20], food insecurity [26] and the professional behaviour of health workers [27]. Efforts to retain PLHIV at risk of attrition from lifelong ART must be prioritized to optimize outcomes [28].

PLHIV who had poor function status at admission had more than seven times the odds of dying during hospitalization compared to those with good function status. Studies from Uganda and Ethiopia using different function assessment tools also revealed associations between poor function status and mortality among hospitalized PLHIV [12, 29]. In other studies, poor function status predicted late presentation of HIV disease [18, 19]. This evidence suggests that assessing functional status among hospitalized PLHIV is crucial for predicting mortality, allowing for identification of high-risk patients who may benefit from rapid interventions to improve outcomes.

PLHIV who had low CD4 + cell counts ≤ 200 cells/µL had 1.68 times the odds of dying during hospitalization compared to those with higher CD4 cell counts. These findings agree with studies from various settings emphasizing the increased risk of mortality associated with low CD4 + cell counts [7, 8], thus underscoring the importance of enhanced CD4 + screening among hospitalized PLHIV. In addition, PLHIV who did not have a documented CD4 + cell count had double the odds of death during hospitalization compared to those with high CD4 + cell counts. While reasons for this observation are not immediately clear, we postulate that increased mortality is linked to the missed opportunities to screen and diagnose important OIs. A recent national survey found suboptimal levels of CD4 + testing among PLHIV, which translated into a missed opportunity to screen 80% of potential TB and Cryptococcal disease patients [30]. Thus, screening and documentation of CD4 + cell counts could be useful amongst hospitalized PLHIV to improve hospital outcomes.

Tuberculosis, Cryptococcal disease and co-infections were highly prevalent in our study. However, both TB and Cryptococcal disease did not predict mortality despite being known predictors of mortality among hospitalized HIV patients [2, 3, 31, 32]. Plausible explanations for this observation might be due to, (a) the declining incidence of these infections underscoring the vast experience gained in investigations and treatment of OIs [5]; (b) the availability of improved rapid diagnostics such as the lateral flow antigen tests which facilitate early diagnosis and treatment of these infections [33–35] or (c) the protective effect of prophylactic treatment such as isoniazid to reduce the incidence of opportunistic infections [36, 37]. These findings re-emphasize the importance of screening, prevention and treatment of opportunistic infections to optimize outcomes among hospitalized PLHIV.

Unlike opportunistic infections, PLHIV who were diagnosed with co-infections or COVID-19 had increased odds of mortality during hospitalization. Several studies agree with these observations where co-infections [3, 4] and COVID-19 infection [38] were associated with increased risk of mortality amongst the hospitalized PLHIV. These findings suggest that co-infections remain an important cause of mortality among hospitalized PLHIV, emphasizing the need for enhanced screening and treatment of such among hospitalized PLHIV.

Hospitalized PLHIV who were diagnosed with liver disease in our study had 1.77 times the odds of dying compared to those without liver disease. These findings agree with other studies that reveal a growing burden of liver disease that predisposes to increased risk of mortality among PLHIV [39–41]. Among the common presentations of liver disease among African PLHIV include drug induced liver injury from anti-TB or ART [42, 43], chronic viral infections [44, 45], liver fibrosis [46, 47], and alcoholic liver disease [46, 47]. Screening measures should be enhanced to enable early detection and treatment of high-risk PLHIV who develop liver disease manifestations particularly those on anti-TB medications (due to risk of drug-induced liver injury) or those with viral hepatitis co-infections.

Hospitalized PLHIV who were diagnosed with chronic lung disease had reduced odds of mortality. While we interpret this finding with a degree of uncertainty, existing literature suggests an increasing burden of chronic lung diseases associated with HIV disease such as post-TB lung disease [48–51], chronic obstructive airway disease [52–54], bronchiectasis [55, 56], asthma exacerbations [57] and altered respiratory microbiome predisposing to chronic airway inflammation [58, 59]. However, data on the survival outcomes of PLHIV diagnosed with chronic lung diseases is scarce, with some studies suggesting an increased risk of mortality with HIV disease [57, 59, 60], contrary to our study findings. Further research to better understand the interaction between HIV disease and chronic lung diseases is warranted.

Likewise, hospitalized PLHIV who were diagnosed with neurologic disorders also had reduced odds of mortality in our study. This observation is also not fully understood but one plausible explanation is the fact that such patients are often transferred to another hospital that provides specialized care for neurologic and mental disorders. These masked the true interactions between HIV disease and the comorbidities observed in our study site. Furthermore, the low prevalence of neurologic disorders in our study could have contributed to the masked interaction with HIV disease. Neurologic disorders have varied manifestations in HIV disease such as HIV-associated cognitive disorders [61, 62], seizure disorders [63] and major depressive disorders [64]. Some studies suggest an increased risk of mortality associated with neurologic disorders amongst PLHIV [65]. These complexities highlight the challenges in the care for PLHIV diagnosed with neurologic disorders in resource poor settings but warrant further research to understand the magnitude and interaction with HIV disease.

PLHIV who were readmitted to hospital also had reduced odds of mortality compared to the new admissions. About one in ten PLHIV in our study were readmitted in our study, a finding slightly lower than recent estimates of 18.8% [66]. The protective effect of hospital readmission in our study is not fully understood and equally warrants further research into understanding this interaction.

### Study limitations

In this large single site study, we identified factors that predicted in-hospital mortality among hospitalized PLHIV in Uganda. However, we acknowledge a few limitations inherent to the study. One, we lacked autopsy reports to confirm the causes of death among the patients, thus potentially missing some key clinical diagnoses [67]. The causes of death were inferred from the clinical diagnoses since autopsies are not routinely done at the hospital due to multiple reasons such as refusal by family members [68, 69]. Secondly, the lack of a control group (e.g. HIV negative subpopulation) limited further analysis and interpretation of the results. Data from an in-hospital HIV support program was used to inform the study findings. Thirdly, the retrospective nature of the study meant some variables could not be assessed such as cognitive function in neurologic disorders. In addition, some variables demonstrated high correlation with others further complicating interpretation of the findings. High multicollinearity was overcome by excluding such variables at multivariate analysis. Fourthly, the lack of a standardized categorization system for clinical diagnoses meant some variables could have been misdiagnosed or misclassified especially for those with similar presentations such as immune reconstitution inflammatory syndromes and infections. Lastly, our findings are limited to a single tertiary-level hospital which may be skewed towards patients requiring super-specialized healthcare services. The findings may not be generalized to the majority who access healthcare from lower-level health facilities [70]. Despite these limitations, the study findings provide valuable lessons for improving outcomes for hospitalized PLHIV.

## Conclusion

One in every four PLHIV is at risk of dying following hospitalization in Uganda. The factors that predicted mortality amongst the hospitalized PLHIV were interruption of antiretroviral therapy, low CD4 + cell counts ≤ 200 cells/µL, unknown CD4 + cell count status, function impairment, co-infections, COVID-19 and liver disease.

There is a need for targeted multisectoral interventions to optimize the care and treatment of hospitalized PLHIV. At an individual level, enhanced HIV testing, early initiation and retention on ART should be prioritized to leverage the benefits of lifelong ART, as well as addressing barriers that hinder timely access to healthcare. At the hospital level, strengthening screening protocols to identify most-at-risk PLHIV such as those with advanced HIV disease or unknown CD4 + cell counts, or function impairment could improve survival through timely interventions such as screening and treatment of co-infections and comorbidities. Patient education on disease prevention particularly regarding the non-communicable diseases such as liver disease, may also help avert poor outcomes following hospitalization. Standardizing health information systems by use of standardized classification tools for clinical diagnoses could improve accurate data interpretation and sharing with relevant stakeholders and health policymakers to better understand disease trends and appropriately allocate resources to respond to emerging healthcare threats.

## Data Availability

The datasets generated and/or analysed during the current study are not publicly available due to the privacy and confidentiality of hospital records but are available from the corresponding author on reasonable request.

## Abbreviations

AOR: Adjusted odds ratio
ART: Antiretroviral therapy
CD4+: Cluster of differentiation four positive
CDC: Centre for Disease Prevention and Control
CNS: Central nervous system
COVID: 19–Coronavirus disease 2019
CRAG: Cryptococcal Antigen
ECOG: Eastern Cooperative Oncology Group
HIV: Human immunodeficiency virus
IQR: Inter quartile range
KNRH: Kiruddu National Referral Hospital
KM: Kilometers
LAM: Lipoarabinomannan antigen
MJAP: Makerere University Joint AIDS Program
MOH: Ministry of Health Uganda
OI: Opportunistic Infections
PEPFAR: President’s Emergency Plan for AIDS Relief
PLHIV: People living with HIV
TB: Tuberculosis
